# Investigation of the Role of AT2 Receptors in the Nucleus Tractus Solitarii of Normotensive Rats in Blood Pressure Control

**DOI:** 10.3389/fnins.2019.00589

**Published:** 2019-06-05

**Authors:** Laura Légat, Ilse J. Smolders, Alain G. Dupont

**Affiliations:** ^1^Department of Pharmaceutical Chemistry, Drug Analysis and Drug Information, Research Group Experimental Pharmacology (EFAR), Center for Neurosciences (C4N), Vrije Universiteit Brussel, Brussels, Belgium; ^2^Cardiovascular Center, Universitair Ziekenhuis Brussel, Brussels, Belgium; ^3^Department of Clinical Pharmacology and Clinical Pharmacy (KFAR), Universitair Ziekenhuis Brussel, Brussels, Belgium

**Keywords:** renin-angiotensin–aldosterone system, angiotensin II type 2 receptor, Compound 21, gamma-aminobutyric acid, nucleus tractus solitarii, mean arterial blood pressure

## Abstract

**Aim:**

The nucleus tractus solitarii (NTS) densely expresses angiotensin II type 2 receptors (AT2R), which are mainly located on inhibitory gamma-aminobutyric acid (GABA) neurons. Central AT2R stimulation reduces blood pressure, and AT2R stimulation in the rostral ventrolateral medulla (RVLM), mediates a hypotensive response through a GABAergic mechanism. We aimed to test the hypothesis that an AT2R mediated inhibition of the GABA release within the NTS might be involved in this hypotensive response, by assessing possible alterations in blood pressure and heart rate, as well as in GABA levels in normotensive Wistar rats.

**Methods:**

*In vivo* microdialysis was used for measurement of extracellular GABA levels and for perfusion of the selective AT2R agonist, Compound 21, within the NTS. Our set-up allowed to determine simultaneously the excitatory glutamate dialysate levels. The mean arterial pressure and heart rate responses were monitored with a pressure transducer.

**Results:**

Local perfusion of Compound 21 into the NTS did not modify blood pressure and heart rate, nor glutamate and GABA levels compared to baseline concentrations. A putative effect was also not unmasked by concomitant angiotensin II type 1 receptor blockade with candesartan. Positive control experiments confirmed that the experimental set up had enough sensitivity to detect a reduction in GABA dialysate levels and blood pressure.

**Conclusion:**

The results did not provide evidence for a role of the AT2R within the NTS in the control of blood pressure, nor for an interaction with local GABAergic signaling in normotensive rats.

## Introduction

The importance of brain angiotensin II (Ang II) and, the Ang II type 1 receptor (AT1R) in the regulation of blood pressure (BP) and sympathetic tone is well established (Guyenet, 2006; Dupont and Brouwers, 2010). Ang II mediated stimulation of AT1Rs in key brain areas for the central control of BP, including the paraventricular nucleus (PVN) and the rostral ventrolateral medulla (RVLM), responsible for the sympathetic drive, increases sympathetic nerve activity and BP (Tagawa and Dampney, 1999; Hu et al., 2002; Li et al., 2006; Dupont and Brouwers, 2010).

The nucleus tractus solitarii (NTS) also plays an important role in central BP control. It is the principal site for the termination of baroreceptor afferents, mediating the inhibitory actions of peripheral baroreceptors on sympathetic outflow (Paton and Kasparov, 1999; Guyenet, 2006). The afferent signals provoked by a BP increase activate NTS excitatory glutamatergic neurons which project to and activate inhibitory GABAergic neurons in the caudal ventrolateral medulla (CVLM), which in turn inhibit the glutamatergic neurons driving the sympathetic tone within the RVLM (Steckelings et al., 2017).

It is well established that brain Ang II modulates the central integration of baroreceptor inputs within the NTS through AT1R stimulation, resulting in attenuation of baroreceptor sensitivity and dampening of baroreceptor reflexes and, hence, elevated BP (Matsumura et al., 1998; Ferguson et al., 2001; Wong et al., 2002; Tan et al., 2005; Yao et al., 2008). This AT1R mediated reduction in baroreceptor feedback seems to be due to a biasing of the transmission between baroreceptor afferents and second-order neurons in the NTS, mediated by increased inhibitory GABAergic neurotransmission (Paton et al., 2001; Guyenet, 2006). The NTS is indeed densely populated with GABAergic neurons exerting an inhibitory influence on the NTS-CVLM pathway (Steckelings et al., 2017), GABAergic nerve terminals (Bailey et al., 2008) as well as with GABA_A_ and GABA_B_ receptors (Zhang et al., 2009). In line with this hypothesis, we recently demonstrated that the Ang II mediated hypertensive response to stimulation of AT1Rs within the NTS was GABA dependent and associated with enhanced GABA release, probably from NTS interneurons (Légat et al., 2019). We postulated that this enhanced release of GABA within the NTS inhibits glutamatergic neurons projecting to the CVLM, thereby reducing the activation of the CVLM inhibitory GABAergic nerves with subsequent disinhibition of the sympathetic driving glutamatergic neurons in the RVLM (Légat et al., 2019). This is also consistent with earlier reports that the GABA_B_ receptor agonist baclofen elicits a pressor response when injected into the NTS (Zhang et al., 2009).

Recent research also revealed the presence of angiotensin II type 2 receptors (AT2R) on neuronal cell bodies and nerve terminals within or close to cardiovascular control centers in the brain (Dai et al., 2016; de Kloet et al., 2016; Steckelings et al., 2017). These findings suggest that AT2R may also be involved in central BP control (Steckelings et al., 2017). Although early functional investigations in rats (Lin et al., 1997; Luoh and Chan, 1998) and heart rate (HR) variability and baroreflex function studies in AT2R disrupted mice (Gross et al., 2002) suggested that brain AT2R might have a tonic inhibitory effect on the baroreceptor reflex, there is now accumulating evidence indicating that brain AT2R may exert antihypertensive actions and restore baroreflex function (Brouwers et al., 2015; de Kloet et al., 2017; Steckelings et al., 2017). AT2R mediated effects often counteract those produced by Ang II acting at the AT1R (de Kloet et al., 2016), and the AT2R is now considered as part of the protective arm of the renin-angiotensin system (Steckelings et al., 2017). This is consistent with our previous observations that intracerebroventricular (ICV) administration of the selective AT2R non-peptide agonist, Compound 21 (C21) (Unger and Dahlof, 2010) reduced BP, increased spontaneous baroreflex sensitivity and inhibited sympathetic outflow in normotensive and spontaneously hypertensive rats (Brouwers et al., 2015). Similar responses to ICV C21 were made by others in conscious normotensive Sprague–Dawley rats and in rats with heart failure (Gao et al., 2011, 2014).

Brain AT2R, are predominantly located on GABAergic neurons (de Kloet et al., 2016; Steckelings et al., 2017) suggesting that the sympatho-inhibitory and BP lowering effect induced by central AT2R activation may involve inhibitory GABAergic mechanisms. In line with this hypothesis, we recently demonstrated in normotensive Wistar rats that local administration through microdialysis of C21 into the RVLM resulted in an AT2R mediated consistent BP lowering effect associated with a significant increase in local GABA concentrations and requiring functional GABA_A_ receptors (Légat et al., 2017). Others also reported that overexpression of AT2R within the RVLM in normotensive rats reduced BP by sympatho-inhibition (Gao et al., 2008). Therefore, the hypotensive response to central administration of C21 appears to be mediated at least in part by stimulation of AT2R within the RVLM. In contrast, local administration of C21 into the PVN did not reduce BP and did not alter local GABA concentrations (Légat et al., 2017).

The NTS densely expresses AT2R which are mainly located on GABA neurons within the NTS (de Kloet et al., 2016), and GABAergic neurons play a dominant role in the regulation of cardiovascular reflexes within the NTS (Potts et al., 2003; Bailey et al., 2008; Dufour et al., 2010).

Taking into account that AT1R stimulation within the NTS increased BP and local release of GABA (Légat et al., 2019), and that brain AT2R and AT1R generally exert opposite effects on BP (Steckelings et al., 2017), we hypothesized that stimulation of AT2R on GABA interneurons within the NTS may act to inhibit local GABA release, thereby reducing the tonic inhibition of the NTS glutamatergic neurons which project to and activate the CVLM neurons. This would be expected to result in disinhibition of these GABAergic CVLM neurons, eventually reducing BP (Steckelings et al., 2017). The objective of the present study was therefore to test this hypothesis by assessing possible BP and HR, as well as glutamate and GABA responses to C21 mediated stimulation of AT2R within the NTS of normotensive Wistar rats.

## Materials and Methods

### Animals

*In vivo* experiments were performed on normotensive male albino Wistar rats (Charles River Laboratories, France) weighing 250–300 g at time of surgery. Animals were housed at constant temperature (22 ± 3°C), a relative humidity of 55 ± 10%, and with a 12 h light-dark cycle with free access to food and water. All procedures were carried out in accordance with the National and European guidelines for animal experimental research and were approved by the Ethical Committee for Animal Experiments of the Faculty of Medicine and Pharmacy of the Vrije Universiteit Brussel, Belgium. All possible steps were taken to reduce the number of animals used and to avoid their suffering.

### Experimental Design, Stereotactic Surgery, *in vivo* Microdialysis, and Mean Arterial Pressure Measurement

Normotensive Wistar rats were first subjected to brain surgery, as described previously (Légat et al., 2017). An intracerebral guide cannula was stereotactically implanted 2 mm above the NTS (AP: -1.30 mm, ML: -12.8 mm, -5.6 mm below dura mater).

After overnight recovery, samples were collected through *in vivo* microdialysis from conscious freely moving rats with a temporal resolution of 20 min and analyzed by liquid chromatography for measurement of glutamate and GABA, as described in detail previously (Smolders et al., 1995; Van Hemelrijck et al., 2005; Légat et al., 2017). Microdialysis should be restricted to a narrow time window (16–48h) in order to reestablish blood brain barrier integrity and preceed gliotic reactions. Based on several studies (Benveniste and Diemer, 1987; Grabb et al., 1998; Schiffer et al., 2006; Sumbria et al., 2011) microdialysis sampling was initiated 24 h after probe implantation.

Experiments started with baseline sampling with modified Ringer’s solution (147 mM NaCl, 2.3 mM CaCl_2,_ 4 mM KCl) for 120 min. Consecutively, one of the treatments: [C21 (0.05 or 0.1 μg/μl/h), candesartan (0.5 or 1.5 ng/μl/h), or C21 (0.05 μg/μl/h) + candesartan (0.5 ng/μl/h)] were dissolved in modified Ringer’s solution and perfused at a flow rate of 2 μl/min through the microdialysis probe for 120 min. Finally modified Ringer’s solution was again perfused for 120 min. L-NAME (0.4 μg/μl/h) was used as a positive control to validate sensitivity of the experimental upset to detect a putative decrease in GABA levels, and clonidine (0.15 mg/μl/h) to validate the sensitivity to detect a putative hypotensive response.

The third day, the same rats were anesthetized using 4% sevoflurane gas and subjected to continuous monitoring of the mean arterial pressure (MAP) and HR, before, during and after perfusion of the same treatments, via cannulation of the right carotid artery using a pressure transducer connected to a monitor (Phillips InetlliVue MP50) as described previously (Légat et al., 2017). The right jugular vein was catheterized for fluid maintenance (0.9% NaCl). The experimental protocol started after a 30 min equilibration period following cannulation. Anesthesia was maintained by 2% sevoflurane administration. First the baseline values were recorded for 30 min before the 2 h administration of the test compounds. Finally, values were recorded under perfusion of Ringer’s solution alone during 30 min.

**FIGURE 1:**
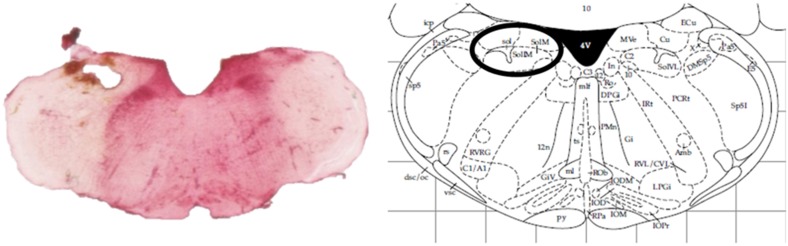
Histological verification of the probe localization within the intermediate NTS by a neutral red staining compared against an anatomic atlas (Paxinos and Watson, 1998).

At the end of the experiments, animals were sacrificed by an overdose of pentobarbital (Dolethal, Vétoquinol, Lure, France). Removed rat brains were kept on 4% paraformaldehyde solutions and probe localization was histologically verified (Figure 1).

### Drugs

The specific and selective AT2R agonist, C21 (K*_i_*: 0.4 nM), was provided by Vicore Pharma AB (Göteborg, Sweden). Nitric oxide synthase inhibitor, *N*(ω)-nitro-L-arginine methyl ester (L-NAME), was purchased from Sigma Aldrich Co. (St. Louis, United States). The AT1R antagonist, candesartan, and alpha (α)_2_-adrenergic receptor agonist, clonidine, were purchased from Tocris Bioscience (Bristol, United Kingdom). Perfusion doses of C21, L-NAME, candesartan and clonidine were based on previous studies (Ogawa et al., 1995; Yang et al., 2005; Mertens et al., 2011; Brouwers et al., 2015).

### Data Analysis and Statistics

Statistical analyses were conducted using Graphpad Prism 6.01 software (Graphpad Software Inc., San Diego, CA, United States) with the α level set at 0.05. Data were expressed as mean ± standard error on the mean (SEM). Microdialysis and MAP levels were expressed as percentages of the baseline levels (considered as 100%) ± SEM. Data were analyzed applying a Friedman test for repeated measures with Dunnett’s *post hoc* multiple comparison test.

## Results

### Lack of AT2R-Mediated Changes on Neurotransmitter Concentrations in the NTS

**FIGURE 2:**
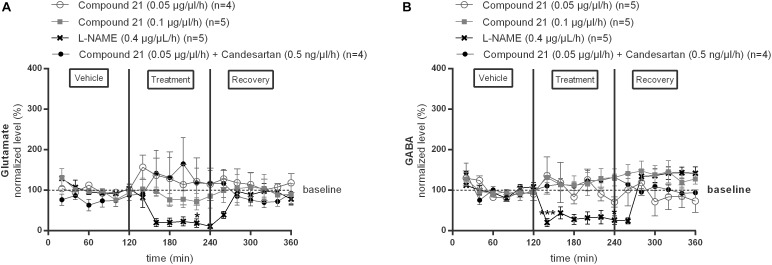
Effect of perfusion of C21 (120–240 min) within the NTS on the extracellular glutamate **(A)** and GABA **(B)** concentrations in normotensive freely moving Wistar rats. Baseline values for glutamate and GABA were respectively, 262 ± 80 and 9 ± 8 nM (C21 0.05 μg/μl/h); 327 ± 270 and 19 ± 8 nM (C21 0.1 μg/μl/h). Baseline values for glutamate and GABA during L-NAME (0.4 μg/μl/h) and C21 (0.05 μg/μl/h) + candesartan (0.5 ng/μl/h) perfusion were respectively, 247 ± 87 and 15 ± 8 nM; 614 ± 274 and 10 ± 8 nM. Data are presented as the mean percentage of baseline levels ± SEM. Statistical analysis is performed using the Friedman test for repeated measures with Dunnett’s *post hoc* multiple comparison. Local L-NAME (0.4 μg/μl/h) perfusion significantly decreased glutamate at time point 220 min (^∗^*p* < 0.05) and GABA levels at time point 140 min (^∗∗∗^*p* < 0.001) and 240 min (^∗^*p* < 0.05) compared to mean baseline values.

We investigated the effect of local C21 perfusion in two different doses (0.05 or 0.1 μg/μl/h) within the NTS on extracellular glutamate (Figure 2A) and GABA (Figure 2B) levels. Extracellular neurotransmitter concentrations were monitored under perfusion of modified Ringer’s solution (0–120 min; 240–360 min) as well as during administration of C21 (120–240 min) dissolved in modified Ringer’s solution. No changes from baseline concentrations were seen for glutamate (Figure 2A; *n* = 4–5) nor GABA (Figure 2B; *n* = 5) levels during C21 perfusion within the NTS (*n* = 5; Figure 2). Co-perfusion of C21 (0.05 μg/μl/h) with candesartan (0.5 ng/μl/h), performed to explore whether AT1R blockade could unmask a putative effect on GABA release, or perfusion of candesartan alone (0.5 or 1.5 ng/μl/h) (data not shown), did not modify glutamate nor GABA dialysis levels (Figure 2A,B; *n* = 4).

In contrast, local perfusion of L-NAME (0.4 μg/μl/h) within the NTS significantly decreased glutamate at time point 220 min (^∗^*p* < 0.05; Figure 2A; *n* = 5) and GABA levels at time point 140 min (^∗∗∗^*p* < 0.001) and 240 min (^∗^*p* < 0.05; Figure 2B; *n* = 5) compared to mean baseline values, indicating that the experimental method was able to detect a decrease in these extracellular amino acid levels.

### Lack of Hypotensive Response to C21 Perfusion Into the NTS

**FIGURE 3:**
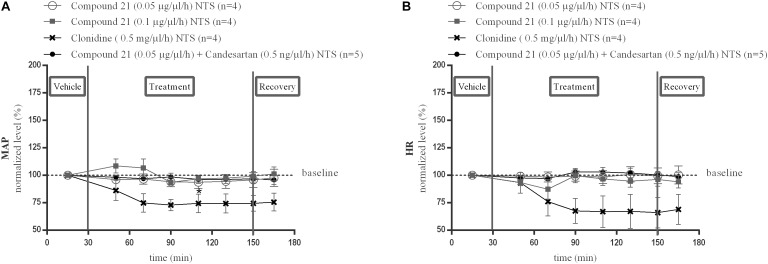
Effect of perfusion of C21(120–240 min) within the NTS on MAP **(A)** and HR **(B)**. Baseline MAP and HR levels were respectively, 86 ± 4 mmHg and 367 ± 35 bpm (C21 0.05 μg/μl/h); 93 ± 4 mmHg and 398 ± 42 bpm (C21 0.1 μg/μl/h). Baseline MAP and HR levels during clonidine (0.15 mg/μl/h) and C21 (0.05 μg/μl/h) + candesartan (0.5 ng/μl/h) perfusion were respectively, 90 ± 3 mmHg and 429 ± 72 bpm; 93 ± 7 mmHg and 404 ± 30 bpm. Data are presented as the mean percentage of baseline levels ± SEM. Statistical analysis is performed using the Friedman test for repeated measures with Dunnett’s *post hoc* multiple comparison. Local perfusion of clonidine (15 mg/μl/h) significantly decreased MAP levels at time point 110 min (^∗^*p* < 0.05) compared to mean baseline values.

Local perfusion of C21 into the NTS at two different doses (0.05 or 0.1 μg/μl/h) did not change MAP (Figure 3A; *n* = 4) or HR (Figure 3B; *n* = 4) compared to baseline measurements. Perfusion of candesartan alone (0.5 or 1.5 ng/μl/h) (data not shown) or co-perfusion of C21 (0.05 μg/μl/h) with candesartan (0.5 ng/μl/h) did also not modify MAP (Figure 3A; *n* = 5) or HR (Figure 3B; *n* = 5).

Local perfusion of clonidine (0.5 mg/μl/h) within the NTS, used as a positive control, did significantly decrease MAP at time point 110 min (^∗^*p* < 0.05; Figure 3A; *n* = 4) and tended to decrease HR (Figure 3B; *n* = 4) compared to mean baseline values.

## Discussion

The NTS is one of the key areas in the dorsal medulla oblongata involved in the central regulation of BP through modulation of sympathetic outflow and baroreceptor function (Guyenet, 2006). The NTS processes the incoming signals from the baroreceptors and is indirectly connected with the RVLM via inhibitory GABA neurons located in the CVLM (Guyenet, 2006). In normal physiological circumstances, in the absence of exogenous Ang II administration, an increase in BP activates baroreceptors located in the vessel wall of the arteria carotis and the aortic arch. The baroreceptor afferents activate NTS efferent glutamatergic neurons which connect to and activate the GABAergic neurons within the CVLM. These GABAergic nerve terminals originating in the CVLM inhibit the activity of the excitatory glutamatergic neurons within the RVLM that drive the sympathetic tone. Hence, the NTS neurons inhibit the RVLM indirectly through the activation of these inhibitory GABAergic nerve terminals originating in the CVLM (Guyenet, 2006). The NTS itself is also densely populated with inhibitory GABAergic (inter)neurons exerting an inhibitory influence on the NTS-CVLM pathway (Steckelings et al., 2017). Local GABA release from these neurons within the NTS can be expected to increase inhibitory GABAergic neurotransmission and reduce the activity of glutamatergic NTS neurons and, subsequently, the activation of the inhibitory GABA neurons in the CVLM, leading to less suppression of the RVLM neurons that drive the sympathetic tone. The final result is inhibition of baroreceptor afferent input signals triggered by increased BP and less suppression of sympathetic tone or, in other words, dampening of the baroreflex and increased BP.

It is well established that baroreceptor function is dampened by Ang II mediated local AT1R stimulation within the NTS, an effect which was thought to be mediated by a local release of GABA within the NTS based on indirect evidence (Paton and Kasparov, 1999; Paton et al., 2001; Guyenet, 2006). Using microdialysis experiments, we recently provided direct evidence that local AT1R stimulation within the NTS is associated with an increase in local GABA levels. Furthermore, the pressor response evoked by local AT1R stimulation within the NTS was GABA dependent (Légat et al., 2019). We speculated that the enhanced release of GABA within the NTS inhibits glutamatergic neurons projecting to the CVLM, thereby reducing the activation of the CVLM inhibitory GABAergic neurons with subsequent disinhibition of the sympathetic driving glutamatergic neurons in the RVLM, leading to a BP increase (Légat et al., 2019). Through this GABA dependent mechanism, Ang II can interfere with short term BP fluctuations, but could also lead to a baroreceptor resetting within the NTS resulting in an upregulation of the activity of the RVLM neurons and development of hypertension.

There is increasing evidence that brain AT2R and AT1R exert opposite effects on BP. We and other groups have indeed observed hypotensive responses to central AT2R stimulation associated with sympatho-inhibition and increased baroreflex sensitivity (Gao et al., 2011, 2014; Brouwers et al., 2015; Légat et al., 2017). Moreover, recent evidence suggests that these central cardiovascular responses to AT2R stimulation, at least within the RVLM, are dependent on GABAergic signaling (Légat et al., 2017, 2018; Steckelings et al., 2017).

AT2R are present on cell bodies in the NTS, mainly on GABAergic neurons (de Kloet et al., 2016; Steckelings et al., 2017). It is therefore not unreasonable to hypothesize that selective AT2R stimulation within the NTS could result in an effect opposite to that of AT1R stimulation with reduced release of GABA from NTS interneurons synapsing on the dendrites or cell bodies of neurons projecting to the glutamatergic CVLM neurons, thereby leading to disinhibition of these CVLM neurons, which can therefore fully exert their inhibitory effect on the sympathetic drive mediating glutamatergic neurons of the RVLM. The ultimate result would be an increased baroreceptor sensitivity and reduction in BP.

The results of the present study, however, do not confirm this hypothesis. Indeed, local administration of C21 within the NTS in normotensive rats, in doses similar to the doses of C21 shown in earlier studies to reduce BP after administration within the RVLM, did not reduce local GABA levels, nor BP and HR. In contrast and as expected, local administration of the α_2_-receptor agonist clonidine did reduce BP (Wemer et al., 1982), and local administration of the NOS inhibitor L-NAME did significantly reduce local GABA and glutamate concentrations (Waki et al., 2003), confirming that our experimental set up had enough sensitivity to detect the expected changes. As it could not be excluded that a putative reduction in GABA levels mediated by AT2R stimulation could be masked by endogenous Ang II mediated AT1R stimulation resulting in an opposite response, we repeated the experiments with C21 under AT1R blockade with candesartan. Blockade of AT1R, which also had no effects by itself, did however not unmask a putative AT2R mediated response.

Therefore, we conclude that these results provide no evidence for a role of AT2R within the NTS in the control of BP, nor for an interaction with local GABAergic signaling, at least in normotensive rats and by unilateral AT2R stimulation. These results, obtained in normotensive rats, do not exclude such possible role in for example the hypertensive state or other situations associated with increased reflex sympathetic tone, such as heart failure. The improvement in baroreceptor sensitivity which we observed in an earlier study after ICV administration of C21, was indeed greater in hypertensive rats than in normotensive rats (Brouwers et al., 2015). We also found no evidence in previous studies for an involvement of AT2R within the PVN, but we did demonstrate consistent GABA dependent hypotensive responses to C21 when perfused within the RVLM (Légat et al., 2017). We therefore conclude that the hypotensive response to central administration of C21 predominantly originates from stimulation of AT2R located within the RVLM in normotensive rats, as previously demonstrated.

## Data Availability

The raw data supporting the conclusions of this manuscript will be made available by the authors, without undue reservation, to any qualified researcher.

## Ethics Statement

All procedures were carried out in accordance with the National and European guidelines for animal experimental research and were approved by the Ethical Committee for Animal Experiments of the Faculty of Medicine and Pharmacy of the Vrije Universiteit Brussel, Belgium. All possible steps were taken to reduce the number of animals used and to avoid their suffering.

## Author Contributions

LL performed the research and wrote the research manuscript. IJS and AGD designed the research study and gave critical insights in the research manuscript.

## Conflict of Interest Statement

The authors declare that the research was conducted in the absence of any commercial or financial relationships that could be construed as a potential conflict of interest.
